# How psychological resilience shapes adolescents’ sports participation: the mediating effect of exercise motivation

**DOI:** 10.3389/fpsyg.2025.1546754

**Published:** 2025-04-09

**Authors:** Hang Hu, Bo Peng, Weisong Chen, Hongshen Wang, Ting Yu

**Affiliations:** ^1^Sports Training Academy, Chengdu Sport University, Chengdu, Sichuan, China; ^2^Jingshan Primary School, Changshou, Chongqing, China

**Keywords:** psychological resilience, exercise motivation, sports participation, adolescents, demographic differences, structural invariance

## Abstract

**Objective:**

This study aimed to examine the relationships among psychological resilience, exercise motivation, and sports participation in adolescents, with a focus on demographic differences, the mediating role of exercise motivation, and structural invariance across gender.

**Methods:**

A total of 2,588 adolescents from grades 7 to 12 were recruited using stratified random sampling, ensuring representation across school levels and rural–urban residence. Demographic differences were analyzed using independent sample *t*-tests and one-way ANOVA. Structural equation modeling (SEM) was employed to conduct mediation analysis and multi-group invariance testing.

**Results:**

Significant demographic differences were observed. Males reported higher levels of psychological resilience, exercise motivation, and sports participation compared to females (*p* < 0.001). High school students demonstrated greater psychological resilience and exercise motivation, whereas middle school students exhibited higher sports participation. Rural adolescents outperformed urban counterparts across all key variables (*p* < 0.001), highlighting the influence of environmental factors on adolescents’ physical activity. The mediation analysis confirmed that exercise motivation significantly mediated the relationship between psychological resilience and sports participation, with indirect effects accounting for 60.26% of the total effect (*p* < 0.001). Model fit indices (CFI, TLI, RMSEA, SRMR) demonstrated a good model fit, supporting the validity of the proposed relationships. Structural invariance testing further indicated consistent relationships across genders, affirming the robustness and generalizability of the model.

**Conclusion:**

These findings highlight the importance of psychological resilience and exercise motivation in promoting adolescents’ sports participation. The study provides empirical evidence and practical insights for designing targeted interventions to support diverse demographic groups and enhance long-term physical activity engagement among adolescents.

## 1 Introduction

Adolescent sports participation has been widely recognized for its positive effects on physical and mental health (Yilong and Haibo, 2022). However, the psychological factors influencing adolescents’ sports participation behaviors remain underexplored. In recent years, psychological resilience, as an adaptive resource that enables individuals to cope with adversity (Lianqiong et al., 2024), has gained significant attention in academia. Studies have shown that psychological resilience not only helps adolescents cope with life challenges but may also influence their exercise motivation and sports participation behaviors (Patricia et al., 2015; Yacong et al., 2021). Although extensive research has explored the relationship between psychological resilience and factors such as mental health and academic performance (Yueyi et al., 2023; Jing et al., 2023; Qiaolan et al., 2011; Xiayun et al., 2024), systematic investigations into how psychological resilience influences sports participation through exercise motivation remain limited.

Exercise motivation is a key psychological factor affecting adolescents’ sports participation (Qurban et al., 2019). Previous studies have confirmed that motivation serves as the core driving force behind individuals’ engagement in sports activities, directly determining the occurrence and persistence of sports behaviors (Park et al., 2014; De Meester et al., 2016; Tušak et al., 2022). Especially during adolescence, exercise motivation is often influenced by psychological traits such as self-efficacy and emotional regulation (Cetinkalp and Turksoy, 2011; Ke et al., 2023; Bertills et al., 2018). As a positive psychological resource, psychological resilience enhances emotional regulation abilities (Wang et al., 2016; Mota et al., 2021), improves self-efficacy, and fosters a proactive attitude toward overcoming challenges. These psychological traits, in turn, significantly enhance exercise motivation, thereby increasing the frequency and sustainability of sports participation (Jiaming, 2024). Although some studies suggest that psychological resilience may indirectly influence sports participation through exercise motivation, direct empirical support for this mediation effect remains limited. Some research has indicated that motivation serves as a crucial mechanism linking personality traits to behavioral outcomes (Miao et al., 2024; Wu et al., 2021), but few studies have systematically examined whether exercise motivation mediates the relationship between resilience and sports participation. Given the critical role of exercise motivation in maintaining sports engagement, investigating whether it mediates the effects of psychological resilience on sports participation is crucial for understanding the psychological mechanisms underlying adolescent sports behavior.

Although some studies have explored the relationship between psychological resilience and sports behaviors (Griciūtė, 2016; Wiedenman et al., 2023), most research has focused on the direct effects of psychological resilience on mental health or academic performance, with limited analysis of how psychological resilience influences sports participation through exercise motivation (Jing et al., 2023; Qiaolan et al., 2011). Furthermore, most existing studies remain descriptive in nature (Min and Qian, 2024; Qiongyang and Wei, 2024), lacking in-depth exploration of the mechanisms through which psychological resilience influences sports participation via exercise motivation. These research limitations highlight the need for a more structured investigation into the mediating role of exercise motivation in the relationship between psychological resilience and sports participation, in order to reveal the specific psychological processes underlying this relationship.

This study aims to fill this research gap by constructing and testing a mediation model in which psychological resilience serves as the predictor variable, exercise motivation as the mediator, and sports participation as the outcome variable, thereby uncovering the underlying mechanisms. Unlike previous studies that primarily focus on the direct effects of psychological resilience on sports participation, this study further investigates how psychological resilience indirectly influences sports participation through exercise motivation, offering a more comprehensive understanding of this process. Additionally, this study examines whether the proposed model applies across different gender groups. Prior research has found that males tend to score higher than females in psychological resilience, exercise motivation, and sports participation (Ramolale et al., 2021), yet there is still a lack of research examining whether the relationships among these variables remain stable across genders. To ensure the robustness and generalizability of the findings, this study employs multi-group structural equation modeling (SEM) to empirically test whether the relationships among psychological resilience, exercise motivation, and sports participation exhibit gender invariance.

By systematically examining the mediating role of exercise motivation, this study not only contributes to theoretical advancements but also provides practical implications. The findings provide empirical support for the theoretical proposition that psychological resilience influences sports participation not only directly but also through its effects on motivation. Furthermore, this research offers valuable insights for educators, policymakers, and sports psychologists in designing interventions aimed at enhancing exercise motivation and psychological resilience to promote long-term sports participation. By identifying exercise motivation as a key mechanism linking resilience and sports participation, this study emphasizes the importance of developing targeted strategies to support adolescents from diverse demographic backgrounds in maintaining sustained participation in sports and physical activities.

## 2 Literature review and research hypotheses

### 2.1 The relationship between psychological resilience and adolescents’ sports participation

Psychological resilience refers to an individual’s ability to effectively adapt and recover from stress, challenges, or adversity (Lianqiong et al., 2024). For adolescents, psychological resilience not only helps alleviate emotional distress but also enhances their belief in their own abilities, promoting positive coping strategies when facing challenges (Liebenberg and Scherman, 2021; Cui and Xie, 2021). In the context of sports, adolescents often encounter physical challenges, emotional fluctuations, and pressures from peer interactions. Higher psychological resilience enables them to better regulate these stressors, thereby increasing the positivity and persistence of their participation (Moljord et al., 2014; Lin et al., 2024; Guo and Liang, 2023).

Psychological resilience influences adolescents’ performance in sports by enhancing their emotional regulation abilities and self-efficacy (Xuliang et al., 2023; Peng et al., 2023). Emotional regulation helps them mitigate negative emotions arising from failures or setbacks, boosting their confidence and motivation for sustained engagement in sports (Sumei and Xiaoyan, 2024). Additionally, psychological resilience strengthens adolescents’ self-efficacy, which is their belief in successfully completing sports tasks. This belief further drives their motivation to participate in sports activities (Cetinkalp and Turksoy, 2011).

Building on this foundation, psychological resilience may facilitate sustained sports participation among adolescents by optimizing emotional responses, improving coping strategies, and strengthening self-efficacy (Sumei and Xiaoyan, 2024; Tao and Guoli, 2021). While the impact of psychological resilience on adolescents’ mental health has been widely acknowledged (Liu, 2024; Mesman et al., 2021), its specific role and mechanisms in shaping sports participation behaviors require further exploration to clarify its potential contributions to sports engagement (Yueyi et al., 2023; Jing et al., 2023).

Based on this understanding, we propose the following hypothesis:

*Hypothesis1 (H1):* Psychological resilience is positively associated with adolescents' sports participation.

### 2.2 The relationship between psychological resilience and exercise motivation

Psychological resilience refers to an individual’s ability to effectively regulate emotions, maintain a positive psychological state, and recover to normal functioning when faced with stress and adversity (Lianqiong et al., 2024). Higher psychological resilience enhances adolescents’ emotional regulation and self-efficacy during sports, thereby boosting their motivation to participate in physical activities (Moljord et al., 2014; Lin et al., 2024). Research shows that psychological resilience helps individuals cope with difficulties and challenges in sports, reduces anxiety and frustration, and increases their persistence in exercising despite setbacks (Lu et al., 2013; Shuming and Hongbo, 2023). Additionally, psychological resilience fosters self-efficacy—the confidence in one’s ability to complete tasks—which further stimulates exercise motivation (Yiqiu et al., 2020). Adolescents with high psychological resilience are better equipped to handle difficulties and failures in sports, maintaining a consistent willingness to participate (Xinghong, 2012).

Psychological resilience also plays a crucial role in regulating emotional responses, enabling individuals to sustain positive emotions and a healthy psychological state during sports (Ma, 2021; Ke et al., 2022). This is essential for maintaining and enhancing exercise motivation. Adolescents with high psychological resilience are more likely to view physical activities as opportunities for self-improvement and psychological adjustment (Yunling et al., 2023), thus exhibiting stronger intrinsic motivation and continuing their engagement in sports. Although previous studies indicate that psychological resilience has a positive impact on exercise motivation, the mechanisms underlying this relationship remain unclear.

Based on this understanding, we propose the following hypothesis:

*Hypothesis2 (H2):* Psychological resilience is positively associated with exercise motivation.

### 2.3 The relationship between exercise motivation and adolescents’ sports participation

Exercise motivation, especially intrinsic motivation, serves as a critical driving force for adolescents’ participation in sports activities. It typically refers to engaging in physical activities for the enjoyment of the process, improving self-efficacy, or achieving personal goals (Yunling et al., 2023; Guangbin and Wenjun, 2011). The strength of intrinsic motivation is closely related to the persistence of adolescents in sports activities (Liu et al., 2017). When adolescents are motivated by internal satisfaction, they tend to exhibit higher participation frequency and quality (Bing and Xiaoli, 2024; Xuejuan et al., 2024). In contrast, extrinsic motivation (e.g., seeking social recognition or external rewards) can temporarily increase participation (Yimin and Xiang, 2017), but its long-term impact on sports participation is relatively limited.

The influence of exercise motivation on adolescents’ sports participation is primarily achieved through enhancing their self-efficacy, emotional regulation abilities, and interest in sports (Moljord et al., 2014; Lin et al., 2024). High exercise motivation encourages adolescents to overcome difficulties and challenges in physical activities, improving their persistence and proactive engagement (Lin et al., 2019). Additionally, individuals with strong exercise motivation typically enjoy the process of physical activity, improving their emotional states and fostering positive exercise habits (Yujiang, 2015). For example, when adolescents participate in sports driven by intrinsic motivation, they not only view exercise as a means of self-improvement but also experience joy during the process, further strengthening their motivation for continued participation (Barnett et al., 2013).

Although existing studies have demonstrated that exercise motivation significantly influences sports participation (Yaffe and Levental, 2024; Jaakkola et al., 2016), there is a lack of systematic exploration of how, specifically, exercise motivation enhances the sustainability and depth of sports activities among adolescents, particularly under varying contextual conditions.

Based on this understanding, we propose the following hypothesis:

*Hypothesis3 (H3):* Exercise motivation is positively associated with adolescents' sports participation.

### 2.4 The role of exercise motivation in the relationship between psychological resilience and adolescents’ sports participation

The connection between psychological resilience and adolescents’ sports participation through exercise motivation remains an underexplored area. Psychological resilience, as an individual’s capacity to positively adapt to stress and challenges (Lianqiong et al., 2024), may be linked to sports participation, but the underlying mechanisms of this relationship are not yet fully understood. Existing studies suggest that psychological resilience may indirectly influence sports participation behaviors through a psychological driving force (Xiaoqiang and Fen, 2019), with exercise motivation posited as a potential mediator.

Exercise motivation reflects the driving factors behind individuals’ engagement in sports activities, particularly intrinsic motivation (e.g., interest and enjoyment of sports), which has been shown to closely correlate with the sustainability of sports participation (Kekun, 2012; Guangbin and Wenjun, 2011). Research indicates that adolescents with high psychological resilience are more likely to experience positive emotional states and behavioral tendencies when facing challenges in sports activities (McDermott et al., 2020), which may promote long-term commitment to physical activities. However, this potential pathway has not been systematically validated.

Current literature on adolescents primarily focuses on the direct relationship between psychological resilience and sports behaviors (Yajie et al., 2021; Xinghong, 2012), with limited exploration of how exercise motivation connects psychological traits to behavioral outcomes. This research gap highlights the need for further investigation into the mediating role of exercise motivation in the relationship between psychological resilience and sports participation. Such exploration could uncover deeper psychological and behavioral dynamics and provide theoretical support for interventions targeting adolescent sports engagement.

Based on this understanding, we propose the following hypothesis:

*Hypothesis4 (H4):* Exercise motivation mediates the relationship between psychological resilience and adolescents' sports participation.

### 2.5 Construction of the comprehensive theoretical hypothesis model

**Figure 1:**
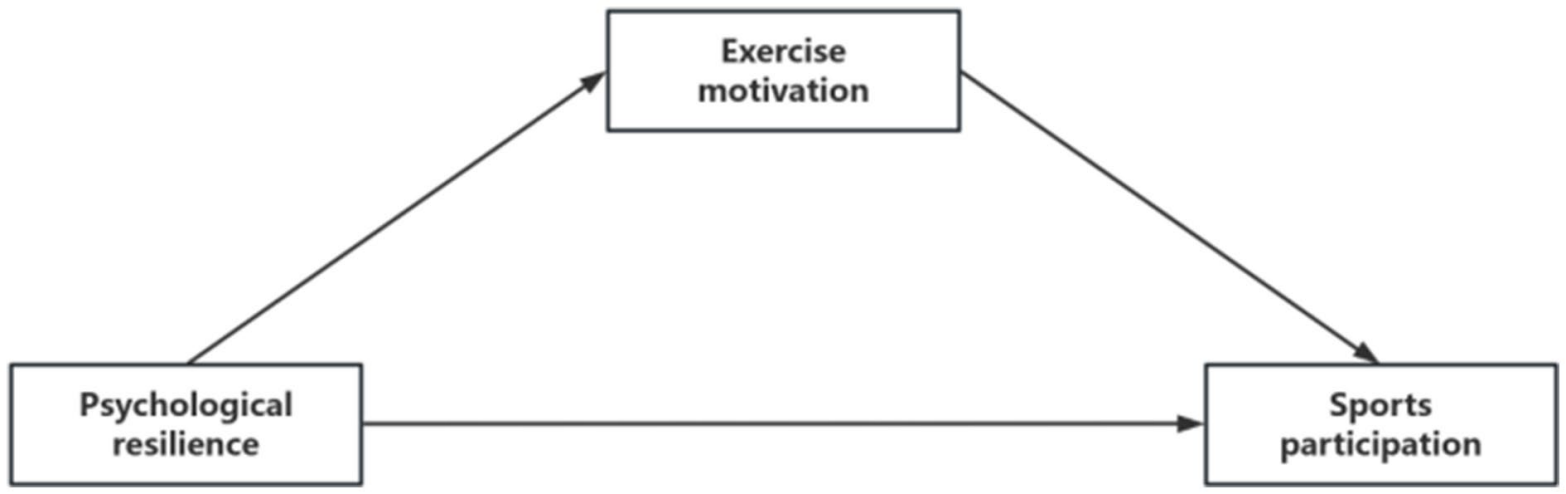
Theoretical model of the relationship between psychological resilience and adolescents’ sports participation: the mediating effect of exercise motivation.

Based on the literature review and the proposed hypotheses, this study constructs a theoretical hypothesis model, as shown in Figure 1. The model aims to integrate existing research findings, refine the relationships between hypotheses, and extend the theoretical framework to provide a more comprehensive understanding of the research topic.

## 3 Materials and methods

### 3.1 Research subjects and data

#### 3.1.1 Rationality of sample size

To ensure the scientific rigor of the research design and the reliability of the results, the required sample size was carefully estimated using both GPower analysis and empirical rules commonly applied in social science research.

First, a power analysis was conducted using G Power 3.1 software for multiple regression analysis. Assuming a medium effect size (f^2^ = 0.15), a significance level of *α* = 0.05, and statistical power (1 − *β*) = 0.80, the results indicated that at least 100 participants were required to detect statistically significant effects. This ensures that the relationships between psychological resilience, exercise motivation, and sports participation can be evaluated accurately within the predefined statistical power.

Second, according to empirical rules in social science research, the recommended sample size should be 10 to 15 times the total number of questionnaire items. The measurement tools in this study included the Psychological Resilience Scale (10 items), Sports Participation Scale (3 items), and Exercise Motivation Scale (28 items), totaling 41 items. Therefore, the recommended sample size range was 410 to 615 participants (i.e., 41 × 10 to 41 × 15). This method further ensures the scientific validity and representativeness of the sample in the context of questionnaire design.

By integrating the two estimation methods, the minimum sample size requirement for this study was determined to be 410 participants, with an ideal range of 410 to 615 participants. In the actual data collection process, to enhance the statistical power and external validity of the study, data were collected from 2,588 adolescents, far exceeding the minimum requirement. This large-scale sample not only strengthens the reliability of the analysis of relationships among the study variables but also improves the stability, generalizability, and representativeness of the findings within the population.

#### 3.1.2 Participant selection process

To ensure representation across different regions and demographic groups, this study adopted a stratified random sampling technique to minimize selection bias. The sampling framework included students from grades 7 to 12 across 15 provinces and municipalities in China. The participants of this study were students from grades 7 to 12, covering junior high school (grades 7 to 9) and senior high school (grades 10 to 12). Elementary school students (grades 1 to 6) were excluded due to their limited comprehension abilities. To ensure sample representativeness, this study employed a stratified random sampling method, stratifying by school level (junior and senior high school), gender, and family location (urban and rural) to ensure balanced inclusion across groups.

Stratification by region: First, China was divided into five geographic strata based on its socioeconomic and cultural diversity. Three provinces or municipalities were randomly selected from each stratum to ensure balanced regional representation (see Table 1 for details of the selected regions and provinces). Next, within the selected provinces or municipalities, students from grades 7 to 12 were randomly sampled, with the number of students adjusted proportionally according to the distribution of schools in each region, ensuring that the sample accurately reflected the overall characteristics of students from different regions. Finally, within each selected school, one to two classes were randomly chosen, and all students within the selected classes were invited to participate in the study.

**Table 1:** Stratification by region and province in the sampling frame.

Region	Province
Eastern China	Shanghai, Jiangsu, Shandong
Central China	Henan, Hubei, Hunan
Western China	Sichuan, Guizhou, Chongqing
Southern China	Guangdong, Guangxi, Hainan
Northern China	Beijing, Shaanxi, Liaoning

Participants were required to meet the following inclusion criteria: currently enrolled in grades 7 to 12, able to independently complete the questionnaire, and regularly engaged in physical exercise. During the data cleaning process, invalid or ineligible questionnaires, such as those with significant missing data or fixed-pattern responses, were excluded to ensure the quality and reliability of the data.

#### 3.1.3 Data collection methods

To ensure the validity and reliability of the data, this study employed a rigorous standardized procedure for data collection. The specific process is as follows:

**Training of data collectors:** Before distributing the questionnaires, the research team provided systematic training to physical education teachers and class teachers. The training covered the explanation of the research objectives, the importance of random sampling, and the specific procedures for standardized questionnaire distribution. Through guidance and scenario simulations, the data collectors were prepared to administer and supervise the questionnaire process consistently, minimizing potential human bias that could affect data quality.
**Questionnaire distribution and completion:** The questionnaires were distributed during regular school hours in a quiet, distraction-free environment, with physical education teachers and class teachers supervising the process to ensure students completed the forms independently without external interference. Upon completion, the research team collected the questionnaires immediately and sealed them uniformly to ensure the timeliness and accuracy of the data, preventing any information leakage.
**Confidentiality and ethical approval:** This study strictly adhered to the principles outlined in the Declaration of Helsinki and complied with ethical guidelines issued by relevant national and institutional authorities. The research protocol was formally reviewed and approved by the Ethics Committee of Chengdu Sport University (Approval Number: CTYLL2024018) to ensure compliance with ethical research standards. Given that this study involved minor participants, ethical considerations were prioritized to safeguard their rights and well-being. Before the study commenced, dual informed consent was obtained: verbal assent from the students and written consent from their parents or legal guardians. In certain cases, where logistical constraints or difficulties in obtaining written consent were present, the Ethics Committee carefully reviewed and approved exemption requests, ensuring that the study was conducted within an ethically sound framework.
**Data privacy and anonymity:** All data were processed anonymously, with no personally identifiable information collected. The research team strictly adhered to data confidentiality requirements, employing encryption technology for data analysis and storage to protect participants’ privacy and ensure data security. Subsequent data-sharing operations were conducted within a secure, encrypted environment to maintain data integrity and confidentiality.

#### 3.1.4 Data processing

This survey lasted for 5 months, from April 1, 2024, to September 1, 2024. A total of 2,900 questionnaires were distributed to ensure a broad and representative sample. During data processing, strict quality control measures were applied. Invalid questionnaires, including those with missing responses, incorrect answers, or fixed-pattern selections, were excluded. Ultimately, 2,588 valid questionnaires were retained, yielding an effective response rate of 89.24%.

During the screening process, we observed that a higher proportion of invalid responses came from female participants. Initially, the distributed questionnaires achieved a gender ratio of approximately 55.17% male and 44.83% female. However, after removing invalid responses, the final dataset resulted in 60.63% male and 39.37% female. This shift in gender ratio was a consequence of strict data quality criteria rather than a sampling imbalance. Despite this adjustment, the final sample remains statistically robust, far exceeding the recommended thresholds in social science research and G*Power analysis, ensuring strong validity and reliability of the findings.

Detailed respondent information is provided in Table 2.

**Table 2:** The sample information.

Basic information	Category	Frequency	Percentage	Cumulative percentage
Gender	Male	1,569	60.63%	60.63%
	Female	1,019	39.37%	100%
School stage	Middle school	1,318	50.93%	50.93%
	High school	1,270	49.07%	100%
Residence type	Rural	922	35.63%	35.63%
	Urban	1,666	64.37%	100%

### 3.2 Measurements

All measurement tools used in this study were based on established scales that have been validated for reliability and validity through multiple studies. The following are the specific measurement tools and related details:

**Psychological resilience:** Psychological resilience was measured using the Resilience Scale developed by Campbell-Sills and Stein (2007). This scale consists of 10 items designed to assess an individual’s adaptability and recovery ability in stressful or adverse situations. The questionnaire employs a 4-point Likert scale, ranging from 1 (Not at all true) to 4 (Completely true), with higher scores indicating higher levels of psychological resilience. This scale has been widely applied across different age groups and cultural contexts, demonstrating high internal consistency and structural validity (Garrido-Muñoz et al., 2024). In this study, the Cronbach’s *α* coefficient of the scale was 0.946, further confirming its excellent reliability.
**Physical exercise:** Sports participation was assessed using the Sports Participation Scale developed by Deqing (1994). This scale consists of three items measuring the frequency, intensity, and duration of adolescents’ engagement in sports activities. Sports participation was calculated using the formula: Physical exercise = Intensity×(Duration−1) × Frequency. where each component follows a five-level scoring system, with a total possible score of 100. This calculation method allows for a more comprehensive assessment of individual differences in physical activity but also results in a relatively large standard deviation due to variations in exercise frequency, intensity, and duration among participants. Previous studies have demonstrated that this scale exhibits good reliability and validity across different cultures and populations (Yao et al., 2023; Liu and Shi, 2023). In this study, the Cronbach’s α coefficient of the scale was 0.803, indicating an acceptable level of reliability.
**Exercise motivation:** Exercise motivation was measured using the Exercise Motivation Scale developed by Liwei and Zhixiong (2004). This scale consists of 28 items across three dimensions, designed to evaluate individuals’ motivation to participate in sports or physical activities and to uncover the psychological and behavioral factors driving such engagement. A 7-point Likert scale was used, ranging from 1 (Not at all true) to 7 (Completely true), with higher scores indicating stronger exercise motivation. Multiple studies have confirmed the good reliability and validity of this scale across different groups and cultural backgrounds (Zhang, 2015; Zeng and Liu, 2013). In this study, the Cronbach’s *α* coefficient of the scale was 0.962, further demonstrating its high reliability.
**Summary:** Table 3 summarizes the scales used in this study, including authors, number of items, scoring range, and dimensions. The selection of these scales was based on their stability and applicability in relevant research fields, with extensive prior studies supporting their high reliability and validity across different cultural contexts and study populations, ensuring the robustness and reliability of the measurements.

**Table 3:** Scales used in this study.

Scale	Author (Year)	Item quantity	Scoring	Dimensions
Psychological resilience	Campbell-Sills and Stein (2007)	10	4	\
Physical exercise	Deqing (1994)	3	5	Exercise intensity; Duration; exercise Frequency
Exercise motivation	Liwei and Zhixiong (2004)	28	7	Intrinsic motivation; Extrinsic motivation; Amotivation

### 3.3 Data analysis procedures

#### 3.3.1 Common method bias

To assess potential common method bias in the data, Harman’s single-factor test was conducted using principal component analysis. All questionnaire items were loaded onto an unrotated factor solution to examine the proportion of variance explained by a single factor. If the variance explained by a single factor exceeds 40%, it may indicate the presence of common method bias, which could threaten the validity of the study.

#### 3.3.2 Descriptive statistics

Descriptive statistics, including means and standard deviations, were calculated for the three key variables: psychological resilience, sports participation, and exercise motivation. This analysis aimed to reveal the central tendency and variability of the sample, providing a comprehensive overview of the sample’s characteristics and forming the basis for subsequent analyses.

#### 3.3.3 Internal consistency reliability

Cronbach’s α coefficient was used to evaluate the internal consistency of each dimension, examining the reliability of the scales in measuring their intended constructs. A Cronbach’s α value of 0.70 or higher was considered acceptable, while a value above 0.80 indicated high internal consistency. This analysis ensured the stability and consistency of the scales, providing robust support for the scientific rigor and credibility of the study results.

#### 3.3.4 Confirmatory factor analysis

To validate the fit of the measurement model, this study conducted confirmatory factor analysis (CFA) in two stages. First, CFAs were separately conducted for the three constructs—psychological resilience, sports participation, and exercise motivation—to independently test whether their factor structures aligned with expectations. The model fit was evaluated using the following criteria: χ^2^/df < 3.0, CFI > 0.90, TLI > 0.90, RMSEA <0.08, and SRMR <0.08, ensuring that the factor models achieved widely accepted standards of fit.

Second, to evaluate the discriminant validity between constructs, multiple CFA models were tested, comparing the three-factor model with other combined models. A better-fitting model for the three-factor structure indicated that the constructs had good discriminant validity, supporting the independence of the dimensions.

#### 3.3.5 Correlation analysis

Correlation analysis was performed to examine the relationships among psychological resilience, sports participation, and exercise motivation. A significance level of *p* < 0.05 was used to test whether statistically significant correlations existed among the variables. By analyzing the size and direction of correlation coefficients, this step provided preliminary evidence to support the hypothesized relationships in the model.

#### 3.3.6 Structural model fit assessment

The structural model’s overall fit was evaluated using multiple indices to verify whether the structural equation model (SEM) accurately reflected the data characteristics. Evaluation criteria included χ^2^/df < 3.0, CFI > 0.90, TLI > 0.90, SRMR <0.08, and RMSEA <0.08. These indices collectively ensured that the model met widely accepted scientific standards of fit, providing a solid foundation for hypothesis testing and the accuracy of path relationships.

#### 3.3.7 Path analysis

Path analysis was conducted within the SEM framework to systematically examine direct and indirect effects among the variables. The significance of path coefficients (*β* values) was determined at *p* < 0.05. The analysis focused on the direct effect of psychological resilience on sports participation while exploring the indirect pathways mediated by exercise motivation. This rigorous examination of path relationships revealed the interactions among variables, providing empirical support for the study hypotheses.

#### 3.3.8 Effect size testing

Effect sizes for direct and indirect effects were assessed using standardized coefficients. The bias-corrected confidence intervals for indirect effects were calculated through bootstrapping with 2,000 resamples. If the 95% confidence interval did not include zero, the mediation effect was considered statistically significant. This method provided high reliability for the precise testing of mediation effects, reinforcing the scientific validity of the study’s conclusions.

#### 3.3.9 Invariance testing across genders

To examine the consistency of the model across genders, a structural invariance analysis was conducted. By comparing nested models, constraints on measurement weights, structural weights, structural covariances, and structural residuals were gradually introduced to assess the model’s applicability across male and female participants. If the ΔCFI value was less than 0.01, the model was deemed structurally invariant, indicating its stability and suitability for different gender groups.

## 4 Results

### 4.1 Common method bias test

To assess the potential impact of common method bias, a principal component analysis was conducted using Harman’s single-factor test. Five factors with eigenvalues greater than 1 were extracted, with the largest factor accounting for 37.61% of the total variance, which is below the critical threshold of 40%. This indicates that common method bias is not a significant concern in this study, suggesting minimal interference with subsequent analyses.

### 4.2 Descriptive statistics, reliability, and construct validity of the measurement model

Table 4 presents the descriptive statistics, internal consistency reliability, and fit indices for the confirmatory factor analysis (CFA) of the key variables, including psychological resilience, sports participation, and exercise motivation.

**Table 4:** Descriptive statistics, internal consistency reliability, and fit indices for confirmatory factor analysis (CFA) of key variables.

Variable	M	SD	α	CFI	TLI	SRMR	RMSEA (90%CI)
Psychological resilience	2.624	0.831	0.946	0.998	0.997	0.008	0.021 (0.014–0.027)
Sports participation	25.516	22.334	0.803	–	–	–	–
Exercise motivation	4.554	1.309	0.962	0.980	0.978	0.016	0.037 (0.035–0.038)

The mean (M) and standard deviation (SD) values indicate that psychological resilience (M = 2.624, SD = 0.831), sports participation (M = 25.516, SD = 22.334), and exercise motivation (M = 4.554, SD = 1.309) display adequate variability across the sample.

The internal consistency reliability (Cronbach’s *α*) for all constructs exceeds the recommended threshold of 0.70, with values of 0.946 for psychological resilience, 0.803 for sports participation, and 0.962 for exercise motivation, demonstrating excellent internal consistency.

The fit indices for the CFA model confirm the construct validity of the measurement model. Specifically, psychological resilience achieves exceptional fit indices (CFI = 0.998, TLI = 0.997, SRMR = 0.008, RMSEA = 0.021 with a 90% confidence interval [0.014–0.027]), while exercise motivation also demonstrates good model fit (CFI = 0.980, TLI = 0.978, SRMR = 0.016, RMSEA = 0.037 with a 90% confidence interval [0.035–0.038]). These results support the unidimensionality and construct validity of the respective measures.

To further evaluate the structural validity of the key constructs, Table 5 compares alternative factor structures. The three-factor model, which distinguishes psychological resilience (PR), exercise motivation (EM), and sports participation (SP), demonstrates superior fit (χ^2^ = 238.37, df = 101, CFI = 0.995, TLI = 0.994, SRMR = 0.019, RMSEA = 0.023 with a 90% confidence interval [0.019–0.027]). This model significantly outperforms the two-factor model (PR + EM, SP; Δχ^2^ = 1943.86, Δdf = 2, *p* < 0.01) and the one-factor model (PR + EM + SP; Δχ^2^ = 1575.95, Δdf = 3, *p* < 0.01).

**Table 5:** Comparative fit indices for alternative factor structures of key constructs.

Model	Factor	χ2	*df*	△χ2 (△*df*)	CFI	TLI	SRMR	RMSEA (90%CI)
Three-factor model	PR, EM, SP	238.37	101	–	0.995	0.994	0.019	0.023 (0.019–0.027)
Two-factor model	PR + EM, SP	2182.23	103	1943.86 (2)	0.919	0.905	0.080	0.088 (0.085–0.092)
One-factor model	PR + EM + SP	3758.18	104	1575.95 (3)	0.857	0.835	0.095	0.117 (0.113–0.120)

PR, psychological resilience; SP, sports participation; EM, exercise motivation. All △χ2 passed the significance test at 0.01 level.

The results confirm that the three-factor structure provides the best representation of the data, with superior goodness-of-fit indices compared to the alternative models. These findings affirm the distinctiveness of psychological resilience, exercise motivation, and sports participation as separate constructs.

### 4.3 Correlation analysis among key variables

**Figure 2:**
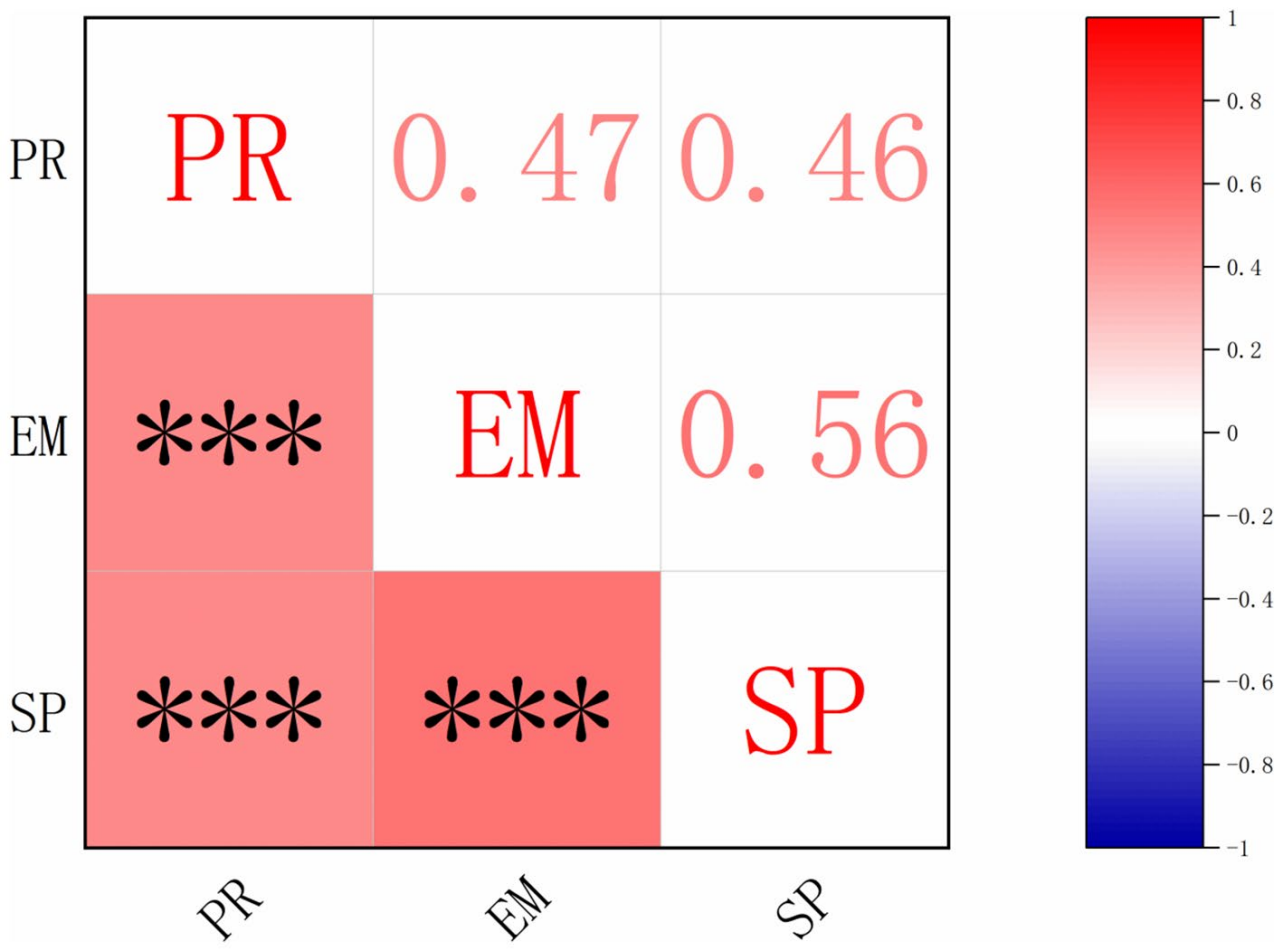
Correlation matrix of key variables. PR, psychological resilience; SP, sports participation; EM, exercise motivation.

The correlation analysis among psychological resilience (PR), exercise motivation (EM), and sports participation (SP) is presented in Figure 2. The results show significant positive correlations among the three key variables, supporting their interrelatedness in the context of adolescent sports participation.

Specifically, psychological resilience is positively correlated with exercise motivation (*r* = 0.47, *p* < 0.001) and sports participation (*r* = 0.46, *p* < 0.001). Additionally, exercise motivation exhibits a strong positive correlation with sports participation (*r* = 0.56, *p* < 0.001).

These findings highlight the foundational role of psychological resilience and its influence on both exercise motivation and sports participation. Furthermore, the strong relationship between exercise motivation and sports participation suggests that motivation acts as a critical mechanism linking resilience to participation behaviors.

The significance levels are denoted in Figure 2, where *p* < 0.001, indicating highly significant associations. The correlation matrix visually illustrates the strength and direction of these relationships, with red shading and larger font sizes reflecting higher positive correlations.

### 4.4 Differences in key variables among demographic groups of adolescents

Table 6 presents the descriptive statistics and *t*-test results for differences in key variables across demographic groups, including gender, grade level, and family location. The findings reveal significant differences in psychological resilience, exercise motivation, and sports participation among different groups of adolescents.

**Table 6:** Descriptive statistics and differences across demographic variables for key variables.

Demographic variables	Category	Psychological resilience		Exercise motivation		Sports participation	
		M	SD	M	SD	M	SD
Gender	Male	2.713	0.821	4.720	1.274	27.892	22.952
	Female	2.487	0.827	4.300	1.322	21.858	20.835
	*t*	6.816		8.066		6.914	
	*p*	0.000		0.000		0.000	
Grade	Middle school	2.541	0.833	4.480	1.320	29.339	23.608
	High school	2.709	0.819	4.631	1.293	21.549	20.187
	*t*	−5.170		−2.936		9.033	
	*P*	0.000		0.003		0.000	
Family location	Urban	2.315	0.794	4.017	1.289	18.421	19.296
	Rural	2.795	0.801	4.852	1.223	29.443	22.930
	*t*	−14.638		−16.066		−12.995	
	*P*	0.000		0.000		0.000	

Male adolescents reported significantly higher levels of physical self-esteem (M = 2.890, SD = 0.612), psychological resilience (M = 2.713, SD = 0.821), exercise motivation (M = 4.720, SD = 1.274), social support (M = 3.699, SD = 0.746), and sports participation (M = 27.892, SD = 22.952) compared to females (*p* < 0.001 for all variables). These results suggest that gender plays a critical role in shaping adolescents’ sports-related behaviors and psychological traits, with males showing advantages in all measured aspects.

High school students exhibited higher levels of psychological resilience (M = 2.709, SD = 0.819), exercise motivation (M = 4.631, SD = 1.293), and physical self-esteem (M = 2.887, SD = 0.610) compared to middle school students (*p* < 0.001). Conversely, middle school students reported greater levels of social support (M = 3.759, SD = 0.743) and sports participation (M = 29.339, SD = 23.608), suggesting that younger adolescents may receive more encouragement and opportunities for physical activity within their social environments.

Adolescents from rural areas reported significantly higher scores in all key variables compared to their urban counterparts (*p* < 0.001). Rural adolescents demonstrated greater physical self-esteem (M = 2.932, SD = 0.593), psychological resilience (M = 2.795, SD = 0.825), exercise motivation (M = 4.852, SD = 1.223), social support (M = 3.708, SD = 0.765), and sports participation (M = 29.443, SD = 22.930). These findings highlight the potential influence of rural environments in fostering adolescents’ physical activity and psychological well-being.

### 4.5 Test results of mediation effects

Table 7 displays the overall fit indices of the structural equation model. The model demonstrates excellent fit (χ2/df = 2.876, CFI = 0.992, TLI = 0.980, SRMR = 0.016, RMSEA = 0.055 [90% CI: 0.039–0.072]), indicating the robustness and adequacy of the model in capturing the relationships among psychological resilience (PR), exercise motivation (EM), and sports participation (SP).

**Table 7:** Questionnaire model fitting indicators.

Model fit	χ2/*df*	CFI	TLI	SRMR	RMSEA (90%CI)
Model	2.876	0.992	0.980	0.016	0.055 (0.039–0.072)

**Figure 3:**
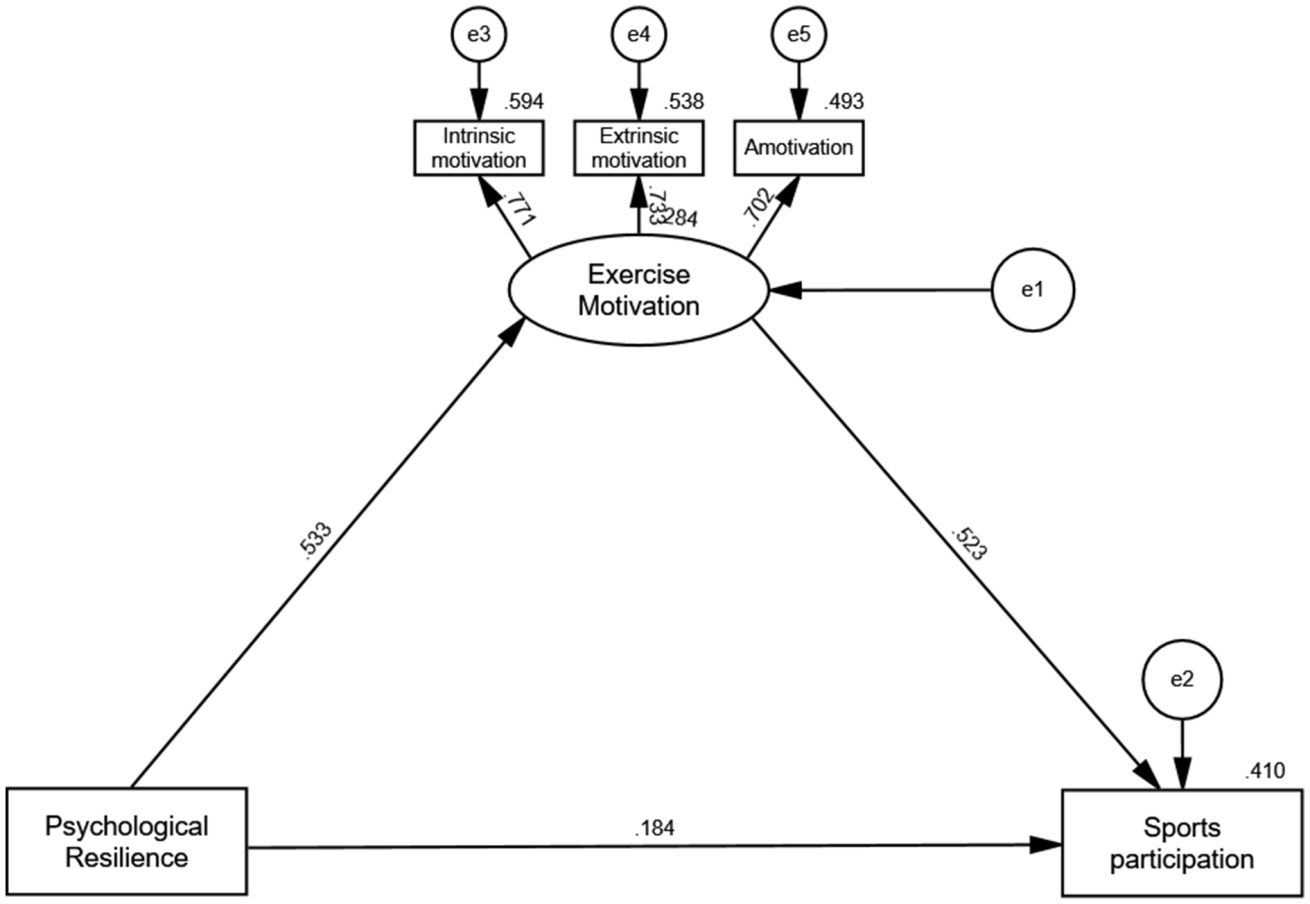
Structural equation model. All paths are significant at the 0.001 level.

Figure 3 illustrates the structural equation model, highlighting the direct and indirect relationships among the key variables. All paths in the model are statistically significant at the 0.001 level, confirming the study’s hypotheses:

Psychological resilience positively predicts exercise motivation (*β* = 0.53, *p* < 0.001). Exercise motivation positively predicts sports participation (*β* = 0.52, *p* < 0.001). Psychological resilience directly predicts sports participation (*β* = 0.18, *p* < 0.001).

Additionally, intrinsic motivation, extrinsic motivation, and amotivation all load significantly onto the construct of exercise motivation, reinforcing its multidimensional nature.

Table 8 presents the total, direct, and indirect effects in the mediation analysis. The results confirm the mediating role of exercise motivation in the relationship between psychological resilience and sports participation:

**Table 8:** Total, direct and indirect effects in the multiple mediator model.

Path	Estimated effect	Boot SE	*p*	Boot LLCI	Boot ULCI	Ratio
Direct effect						
PR → SP	0.184	0.021	0.000	0.144	0.226	39.74%
Indirect effects						
PR → EM → SP	0.279	0.015	0.000	0.249	0.310	60.26%
Total effect	0.463	0.015	0.000	0.432	0.492	100%

PR, psychological resilience; SP, sports participation; EM, exercise motivation. Boot LLCI, the lower bound of the 95% confidence interval. Boot ULCI, the upper limit of the 95% confidence interval (percentile bootstrap method with bias correction). The Bootstrap sample size is set at 2000.

The direct effect of psychological resilience on sports participation is significant (*β* = 0.184, *p* < 0.001), accounting for 39.74% of the total effect.

The indirect effect of psychological resilience on sports participation through exercise motivation (*β* = 0.279, *p* < 0.001) accounts for 60.26% of the total effect.

The total effect (*β* = 0.463, *p* < 0.001) illustrates the combined influence of direct and indirect paths.

The study’s hypotheses are supported as follows:

H1: Psychological resilience is positively associated with adolescents’ sports participation. This is supported by the significant direct effect (*p* < 0.001).
H2: Psychological resilience is positively associated with exercise motivation, confirmed by the strong path coefficient (*p* < 0.001).
H3: Exercise motivation is positively associated with adolescents’ sports participation, evidenced by the significant relationship (*p* < 0.001).
H4: Exercise motivation mediates the relationship between psychological resilience and adolescents’ sports participation. This is supported by the significant indirect effects (*p* < 0.001).

The findings highlight the critical role of exercise motivation as a mediator in the relationship between psychological resilience and sports participation. Psychological resilience positively influences both exercise motivation and sports participation, with exercise motivation acting as a significant pathway to enhance adolescents’ participation in sports.

### 4.6 Testing for structural invariance across gender

Table 9 presents the results of structural invariance testing across gender groups. This analysis assessed whether the relationships among psychological resilience, exercise motivation, and sports participation were equivalent for male and female adolescents. Multiple levels of constraints were applied to the structural model, including measurement weights, structural weights, structural covariances, and structural residuals.

**Table 9:** Testing for structural invariance across gender.

	χ2/*df*	CFI	△CFI	TLI	△TLI	SRMR	RMSEA (90%CI)
Unconstrained	4.431	0.993	–	0.982	–	0.017	0.036 (0.025–0.0419)
Measurement weights	3.513	0.993	0.000	0.987	+0.005	0.017	0.031 (0.021–0.042)
Structural weights	3.352	0.992	−0.001	0.988	+0.006	0.019	0.030 (0.021–0.040)
Structural covariances	3.116	0.992	−0.001	0.989	+0.007	0.019	0.029 (0.019–0.038)
Structural residuals	2.951	0.992	−0.001	0.990	+0.008	0.019	0.027 (0.018–0.037)

The unconstrained model demonstrated excellent fit (χ2/df = 4.431, CFI = 0.993, TLI = 0.982, SRMR = 0.017, RMSEA = 0.036 [90% CI: 0.025–0.0419]), establishing a baseline for comparison. When progressively applying constraints, the changes in fit indices were negligible, as shown below:

Measurement weights model: ΔCFI = +0.000, ΔTLI = +0.005, RMSEA = 0.031. Structural weights model: ΔCFI = −0.001, ΔTLI = +0.006, RMSEA = 0.030. Structural covariances model: ΔCFI = −0.001, ΔTLI = +0.007, RMSEA = 0.029. Structural residuals model: ΔCFI = −0.001, ΔTLI = +0.008, RMSEA = 0.027.

Across all levels of constraints, the changes in CFI (∣ΔCFI∣ ≤ 0.001|) and TLI (∣ΔTLI∣ ≤ 0.008|) were well below the recommended threshold of 0.01, indicating that the constrained models fit the data as well as the unconstrained model.

These results provide strong evidence for structural invariance across gender:

Measurement Invariance: The factor loadings of observed variables were equivalent across genders, indicating that the constructs were interpreted similarly by male and female adolescents.
Structural Invariance: The relationships among psychological resilience, exercise motivation, and sports participation (i.e., path coefficients, covariances, and residuals) did not differ significantly by gender.

The structural invariance testing confirms that the model is robust and generalizable across gender groups. These findings suggest that the roles of psychological resilience and exercise motivation in influencing sports participation are consistent for both male and female adolescents.

## 5 Discussion

### 5.1 Psychological resilience and sports participation

This study supports Hypothesis H1, indicating that psychological resilience is significantly positively associated with adolescents’ sports participation. This result aligns with previous research, suggesting that psychological resilience promotes adaptive behaviors and long-term engagement in sports activities (Hsieh et al., 2023; Deng et al., 2023). However, unlike prior studies that mainly focused on how sports participation enhances resilience, this study provides empirical evidence demonstrating that psychological resilience itself is a critical predictor of sports participation.

From a theoretical perspective, this finding is consistent with psychological resilience theory, which suggests that individuals with higher resilience are better able to cope with adversity and maintain high levels of engagement in meaningful activities, such as sports (Werner and Smith, 1982). Additionally, this study further demonstrates that psychological resilience may facilitate sports participation by enhancing self-efficacy, emotional regulation, and perseverance. This finding also aligns with self-determination theory (SDT), which posits that internal psychological resources can strengthen intrinsic motivation, thereby sustaining long-term sports participation (Mahoney et al., 2014).

### 5.2 Psychological resilience and exercise motivation

This study supports Hypothesis H2, demonstrating that psychological resilience significantly predicts exercise motivation. This finding fills a gap in prior research, which has primarily focused on external incentives (e.g., social support, environmental factors) in shaping exercise motivation (Gillet et al., 2010; Qurban et al., 2019). Instead, this study highlights the critical role of internal psychological factors, particularly psychological resilience, in shaping exercise motivation.

The results suggest that individuals with higher psychological resilience are more confident when facing challenges related to exercise, making them more likely to engage in physical activities and sustain motivation. This implies that enhancing psychological resilience may help stimulate adolescents’ interest in sports and their persistence in regular exercise. Particularly during adolescence, developing coping skills and fostering a positive attitude toward exercise are crucial for maintaining long-term sports participation. Therefore, practical applications should focus on psychological training, goal setting, and positive reinforcement to enhance adolescents’ psychological resilience, thereby promoting their exercise motivation and sustained sports engagement.

### 5.3 Exercise motivation and sports participation

This study supports Hypothesis H3, confirming a significant positive correlation between exercise motivation and sports participation. This result further emphasizes the crucial role of motivation in promoting long-term engagement in sports activities among adolescents. Exercise motivation not only influences whether an individual initiates sports participation but also determines their ability to maintain it over time, which is consistent with existing research (Bing and Xiaoli, 2024; Xuejuan et al., 2024).

Unlike previous studies that predominantly focused on how external incentives (e.g., rewards, peer influence, competition) affect sports participation, this study further highlights the importance of intrinsic motivation. The findings suggest that adolescents with stronger intrinsic motivation are more likely to actively engage in sports and sustain participation even in the absence of external rewards. This result has important implications for sports education and intervention strategies, indicating that fostering intrinsic motivation—such as through personalized goal setting, enhancing autonomy in sports, and making activities more enjoyable—should be prioritized over solely relying on external rewards or compulsory measures to ensure long-term engagement in sports.

### 5.4 The mediating role of exercise motivation

This study supports Hypothesis H4, confirming that exercise motivation mediates the relationship between psychological resilience and sports participation. This finding contributes to the existing literature by demonstrating that psychological resilience not only directly influences sports participation but also indirectly promotes sports behavior by enhancing exercise motivation.

Previous research has mainly focused on the direct relationship between psychological resilience and sports participation, while this study extends the understanding of the mechanisms influencing sports behavior by identifying the mediating role of exercise motivation. This finding underscores that interventions aiming to increase sports participation should not only focus on enhancing psychological resilience but also include strategies to strengthen exercise motivation. Effective approaches may include personalized goal setting, implementing intrinsic reward mechanisms, and fostering a sense of competence in sports performance.

### 5.5 Gender differences

This study finds that male adolescents score significantly higher than females in psychological resilience, exercise motivation, and sports participation, consistent with previous research (Liang-Ting et al., 2015; Van Tuyckom et al., 2010; van Schrojenstein Lantman et al., 2016). However, structural invariance analysis reveals that the relationships between psychological resilience, exercise motivation, and sports participation remain stable across genders, indicating that while males and females differ in absolute levels of these variables, the underlying mechanisms linking resilience, motivation, and participation apply universally.

This finding has important theoretical and practical implications. Previous studies have rarely focused on gender-based comparisons, whereas this study suggests that motivation-based interventions may be equally effective for both genders. Future interventions should consider gender differences in designing motivational strategies, ensuring they are tailored to address barriers specific to each gender while maintaining a consistent framework for fostering resilience and promoting sports participation among both male and female adolescents.

### 5.6 Demographic influences

This study finds that rural adolescents exhibit significantly higher levels of psychological resilience, exercise motivation, and sports participation compared to their urban counterparts. This suggests that rural environments may provide more opportunities for physical activity and stronger social support, promoting both mental and physical well-being. Unlike previous studies that primarily focused on urban adolescents’ sports participation trends, this study provides new empirical evidence on urban–rural disparities in resilience and sports engagement.

Additionally, middle school students demonstrate higher levels of sports participation than high school students but lower levels of psychological resilience and exercise motivation. This finding suggests that as adolescents grow older, increasing academic pressure, time constraints, and shifting social expectations may impact their engagement in sports. These results highlight the need for age-specific sports intervention programs that address the unique challenges faced at different developmental stages to sustain long-term participation in physical activity.

## 6 Theoretical and practical implications

Theoretical Contribution: This study extends psychological resilience theory by identifying exercise motivation as a key mediating variable, enriching the theoretical framework of how psychological resilience influences sports participation. Additionally, it enhances the application of gender studies in sports psychology by demonstrating the consistency of the relationship between psychological resilience and exercise motivation across genders, providing a theoretical basis for developing intervention strategies applicable to both male and female adolescents.

Practical Applications: For educators and policymakers: The study highlights the importance of developing systematic intervention programs to enhance adolescents’ psychological resilience and exercise motivation, including goal setting, resilience training, and intrinsic reward mechanisms. For sports psychologists and coaches: The findings suggest that personalized training strategies, such as improving self-efficacy, increasing intrinsic motivation, and resilience training, may be more effective in promoting long-term sports participation.

## 7 Limitations and future research directions

This study still has certain limitations that need to be addressed in future research. First, it adopts a cross-sectional design, which, while revealing the relationships between psychological resilience, exercise motivation, and sports participation, does not establish causality. Future research should employ longitudinal studies or experimental intervention designs to track changes in psychological resilience, exercise motivation, and sports participation at different time points, thereby verifying their causal pathways. Additionally, the sample in this study is drawn from a specific cultural background, which may limit the generalizability of the findings. Future studies should conduct cross-cultural research to examine the applicability of these relationships among adolescents in different countries or regions and analyze the influence of cultural factors on psychological resilience and exercise motivation to enhance the external validity of the findings.

Furthermore, this study primarily relies on self-reported questionnaires for data collection, which may introduce social desirability bias, as participants might overestimate or underestimate their level of sports participation. Future research should incorporate multiple measurement methods, such as using wearable devices (e.g., accelerometers, heart rate monitors) to obtain objective physical activity data or employing third-party observational assessments from teachers and parents to reduce self-report bias. Additionally, this study did not consider other important factors that might influence sports participation, such as social support, personality traits, and health status. Future research could expand the current model by including these variables to develop a more comprehensive understanding of the factors influencing adolescent sports participation. By addressing these limitations, future studies can further refine the theoretical framework of psychological resilience, exercise motivation, and sports participation and provide more practical insights for sports education and health promotion.

## 8 Conclusion

This study underscores the pivotal roles of psychological resilience and exercise motivation in shaping adolescents’ sports participation. The findings reveal that resilience not only directly enhances sports participation but also exerts a stronger influence through the mediating role of motivation, highlighting motivation as a critical psychological mechanism. Additionally, the structural invariance across gender confirms the robustness and generalizability of the proposed model. These insights provide valuable guidance for designing interventions that foster resilience and motivation, ultimately promoting sustained engagement in sports and supporting adolescents’ holistic development.

## Data Availability

The original contributions presented in the study are included in the article/Supplementary material, further inquiries can be directed to the corresponding author.
