# Supplementary material for: How psychological resilience shapes adolescents’ sports participation: the mediating effect of exercise motivation
Source: Front Psychol. 2025 Apr 9;16:1546754. doi: 10.3389/fpsyg.2025.1546754 (PMC12014653; doi:10.3389/fpsyg.2025.1546754)
Supplement: Supplementary file 1 [file Data_Sheet_1.pdf]

How psychological resilience shapes adolescents' sports participation: the mediating effect of exercise motivation

Dear Student,

Hello! Thank you for taking the time to participate in this survey. This questionnaire aims to explore how psychological resilience influences adolescents' sports participation, with a particular focus on the mediating role of exercise motivation. The goal of this study is to deepen our understanding of the interactions among these factors and provide a scientific foundation for strategies to promote adolescents' physical activity and mental health.

The questionnaire primarily consists of objective questions, and we kindly ask you to answer based on your personal experiences. There are no right or wrong answers, as everyone’s circumstances are different. Please rest assured that all the information you provide will be treated with the utmost confidentiality, will not be disclosed publicly, and will not be used for any personal evaluation. Your honest responses are crucial to this research, so please select the answers that best reflect your actual situation.

We pledge to keep all data collected during this study strictly confidential.

Your participation is vital to the success of this research, and we sincerely thank you for your support and cooperation!

Part 1: Personal Information (Please mark “√” next to the appropriate option)

1. Gender: Male ( ) Female ( )

2. Grade:Middle school ( ) High school ( )

3. Residence type：Rural（ ） Urban（ ）

Part 2: Physical Exercise Level Scale

Please recall your physical exercise over the past month and select the corresponding answer (A, B, C, D, E) in the parentheses based on your actual situation.

1. **What type of physical exercise do you regularly engage in?** ( )

A. Light exercise (e.g., walking, doing light calisthenics)

B. Low-intensity, relaxed exercise (e.g., casual volleyball, table tennis, jogging, tai chi)

C. Moderate-intensity, more vigorous and enduring exercise (e.g., cycling, running)

D. High-intensity exercise with heavy breathing and sweating, but not very prolonged (e.g., badminton, basketball, tennis, soccer)

E. High-intensity and prolonged exercise with heavy breathing and sweating (e.g., racing, aerobic routines, swimming)

2. When engaging in the above intensity of physical activities, how many minutes do you usually spend each time? ( )

A. Less than 10 minutes

B. 11 to 20 minutes

C. 21 to 30 minutes

D. 31 to 59 minutes

E. 60 minutes or more

3. How many times per month do you engage in the above physical activities? ( )

A. Less than once per month

B. 2 to 3 times per month

C. 1 to 2 times per week

D. 3 to 5 times per week

E. Approximately once per day

Psychological Resilience Scale

Serial number	Item	①Completely Disagree ②Mostly Disagree ③Somewhat Agree ④Completely Agree			
1	I can adapt flexibly when changes occur.	1	2	3	4
2	I am capable of handling difficulties when they arise.	1	2	3	4
3	I face challenges with a sense of humor.	1	2	3	4
4	I become stronger through accumulated experiences.	1	2	3	4
5	I recover well after illness or hardship.	1	2	3	4
6	I can achieve my goals even when faced with obstacles.	1	2	3	4
7	I am able to focus and think clearly under pressure.	1	2	3	4

8	I do not feel discouraged by failure.	1	2	3	4
9	I consider myself a strong person when facing challenges in life.	1	2	3	4
10	I am capable of managing unpleasant emotions, such as anger.	1	2	3	4

Exercise Motivation Scale

**Serial number**	**Item**	**①Completely Disagree**<br>**②Disagree**<br>**③Somewhat Disagree**<br>**④Neutral**<br>**⑤Somewhat Agree**<br>**⑥Agree**<br>**⑦Completely Agree**						
1	Because I feel happy during exciting experiences.	1	2	3	4	5	6	7
2	Because sports activities allow me to learn more about the sport I participate in, which brings me a lot of joy.	1	2	3	4	5	6	7
3	I have always had good reasons to exercise, but now I am not sure whether I should continue.	1	2	3	4	5	6	7
4	To experience the joy of discovering new exercise methods.	1	2	3	4	5	6	7
5	I don't know; it seems I cannot achieve success in the sports activities I participate in.	1	2	3	4	5	6	7
6	Because sports activities earn me respect from people I know.	1	2	3	4	5	6	7
7	I think sports activities are one of the best ways to connect with others.	1	2	3	4	5	6	7
8	Because mastering difficult sports techniques gives me a sense of self-satisfaction.	1	2	3	4	5	6	7
9	I believe participating in sports activities is necessary	1	2	3	4	5	6	7

	to maintain good health.							
10	For the reputation of being an athlete.	1	2	3	4	5	6	7
11	Because sports activities are the best way I choose to promote my development in other areas.	1	2	3	4	5	6	7
12	To experience the joy of overcoming some of my weaknesses.	1	2	3	4	5	6	7
13	To feel the excitement of being fully engaged in sports activities.	1	2	3	4	5	6	7
14	Because I must engage in sports activities to achieve a good sense of self.	1	2	3	4	5	6	7
15	To feel satisfaction when I improve my abilities.	1	2	3	4	5	6	7
16	Because the people around me believe a healthy body is very important.	1	2	3	4	5	6	7
17	Through sports activities, I can learn many things that are beneficial for other aspects of life.	1	2	3	4	5	6	7
18	Because when I engage in my favorite sports activities, I feel a strong sense of passion.	1	2	3	4	5	6	7
19	I am not sure now, but I do think my body and sports activities are unrelated.	1	2	3	4	5	6	7
20	Because I feel joy when I complete challenging movements.	1	2	3	4	5	6	7
21	If I don't spend some time on sports activities, I feel uneasy.	1	2	3	4	5	6	7
22	To show everyone how good I am at sports activities.	1	2	3	4	5	6	7
23	To enjoy learning new techniques I haven’t practiced before.	1	2	3	4	5	6	7
24	Because sports activities are the best way for me to maintain good relationships with friends.	1	2	3	4	5	6	7
25	Because I enjoy the feeling of being fully immersed in an activity.	1	2	3	4	5	6	7
26	Because I must exercise regularly.	1	2	3	4	5	6	7
27	To feel the joy of discovering new activity strategies.	1	2	3	4	5	6	7
28	I often tell myself: It seems I cannot achieve the goals I set for myself.	1	2	3	4	5	6	7

The questionnaire is now complete. Thank you for your responses!
